# ﻿Two new species in the fern genus *Lomariopsis* (Lomariopsidaceae) from East Asia

**DOI:** 10.3897/phytokeys.187.77035

**Published:** 2021-12-20

**Authors:** Yi-Hsuan Wu, Chih-Yun Sun, Atsushi Ebihara, Ngan Thi Lu, Germinal Rouhan, Li-Yaung Kuo

**Affiliations:** 1 Institute of Molecular & Cellular Biology, National Tsing Hua University, No.101, Sec. 2, Guangfu Rd., Hsinchu City, Taiwan; 2 Department of Life Science, National Tsing Hua University, No.101, Sec. 2, Guangfu Rd., Hsinchu City, Taiwan; 3 Department of Botany, National Museum of Nature and Science, Amakubo 4–1–1, Tsukuba, Ibaraki, Japan; 4 Department of Biology, Vietnam National Museum of Nature, Vietnam Academy of Science and Technology, 18 Hoang Quoc Viet, Hanoi, Vietnam; 5 Graduate University of Science and Technology, Vietnam Academy of Science and Technology, 18 Hoang Quoc Viet, Hanoi, Vietnam; 6 Institut de Systématique, Evolution, Biodiversité (ISYEB), Muséum national d’Histoire naturelle, CNRS, Sorbonne Université, EPHE, Université des Antilles; CP 39, 57 rue Cuvier 75005 Paris, France

**Keywords:** Cryptic species, independent gametophyte, *Lomariopsisboninensis*, *Lomariopsislongini*, *Lomariopsismoorei*, *Lomariopsisspectabilis*, phylogeny, systematics

## Abstract

Two East Asian *Lomariopsis* (Lomariopsidaceae, Polypodiales) species, *Lomariopsismoorei* and *Lomariopsislongini*, which were previously misidentified as *L.spectabilis*, are here described as new species based on evidence from morphological characters and a molecular phylogeny. The two species differ from the three other described species in East Asia by their venation, pinna shapes, and perine morphology. A phylogeny based on a combined dataset of three chloroplast regions (*rbcL*+ *rps4-trnS* + *trnL-L-F*) showed that *L.moorei* and *L.longini* each formed a well-supported monophyletic group which was distantly related to both *L.spectabilis* and the other morphologically similar East Asian species, *L.boninensis*.

## ﻿Introduction

*Lomariopsis* Fée is the most species rich genus in the fern family Lomariopsidaceae and contains approximately 60 spp., accounting for 85% of the family (PPG I 2016). This genus has a wide distribution in tropical and subtropical regions; there are 15 species in the Neotropics (Moran 2000), nine species in Africa (Roux 2009), 11 species in the islands of the Indian Ocean (Holttum 1939b; Roux 2009; Rakotondrainibe and Jouy 2017), and 12 species in Asia and the Oceanian region (Holttum 1932, 1939a, 1966, 1978). The latest phylogeny of *Lomariopsis* included 24 species (ca. 40% of the species diversity in *Lomariopsis*), but only two species from Asia and the Oceanian region have been sampled (Chen et al. 2017) while the vast majority (ca. 10 species) from these areas have not yet been surveyed. In addition, because gametophytes of *Lomariopsis* species are able to establish as long-lived, asexual colonies in the wild (Watkins and Moran 2019), several species are found as gametophyte-only populations, which is called independent gametophytes (Pinson et al. 2017). In Japan and Taiwan, gametophytes of unknown species have been also reported (Ebihara et al. 2013; Wu et al. in press), a finding which further points out that the efforts of systematics research for Asian *Lomariopsis* remains inadequate, and there might have been undocumented and cryptic species.

To investigate phylogenetically *Lomariopsis* from these poorly sampled areas, we sampled most Asian and Oceanian species, including all species in East Asia where two previously unidentified species were discovered. They both had been misidentified as *L.spectabilis* Mett. One was from Chiayi County in Taiwan and Hainan Island in China, and the other one was from northern Vietnam and west southern China. They are superficially similar to two Asian species, *L.boninensis* Nakai and *L.spectabilis* in morphology. In this study, we presented a new *Lomariopsis* phylogeny supplied with comprehensive East Asian sampling, and reevaluated diagnostic characters leading to the description of these species.

## ﻿Materials and methods

### ﻿Perine morphology and spore number in sporangia

Spores were taken from mature sporangia and fixed on double-sided tape, and then gold coated with a sputter-coater for 1–3 min. Spores were subsequently examined with a tabletop SEM (TM 3000; Hitachi, Ibaraki, Japan).

To examine the spore number per sporangium, at least five mature, unopened sporangia per specimen were collected. These sporangia were broken individually, and we counted the number of spores inside under a stereomicroscope.

### ﻿DNA extraction and chloroplast DNA region sequencing

Twenty-nine samples were included in our molecular phylogenetic study. Voucher information is provided in Appendix 1 (i.e., those samples noted with *). Total DNA extraction was done following the modified CTAB protocol of Kuo (2015). Three chloroplast (cp) regions were amplified and sequenced: *trnL-L-F* (*trnL* gene + *trnL-trnF* intergenic spacer), the gene *rbcL*, and *rps4-trnS* (*rps4* gene + intergenic spacer), which were also used in previous phylogenies of *Lomariopsis* and Lomariopsidaceae (Rouhan et al. 2007; Li et al. 2009; Chen et al. 2017). The primers used for PCR amplification and sequencing were: FernL 1Ir1 (Li et al. 2010) and f (Taberlet et al. 1991) for *trnL-L-F*; rps5 (Nadot et al. 1994) and trnS (Souza-Chies et al. 1997) for *rps4-trnS*; af (Hasebe et al. 1994) and 1379R (Pryer et al. 2001) for *rbcL*. PCR amplifications were prepared in 15 μL reactions each containing 20 ng of genomic DNA, 1× SuperRed PCR Master Mix RED (TOOLS, Newtaipei City, Taiwan) and 0.5 μM of each primer. A typical amplification program began with one initial denaturation step for 5 min at 94 °C then 35 cycles of 1 min at 94 °C, 30 s at 55 °C, and 1 min at 72 °C followed by a final extension of 10 min at 72 °C and was performed on a SimpliAmp Thermal Cycler. PCR products were cleaned using ExoSAP-IT (Thermo Fisher Scientific, Waltham, Massachusetts, USA), and then sequenced with the same PCR primers with an ABI 3730XL (Thermo Fisher Scientific, Waltham, Massachusetts, USA) by the Genomics BioSci. & Tech. company in Taiwan. GenBank accession numbers of the sequences are listed in Appendix 1.

### ﻿Phylogenetic analyses

In total, we sampled 35 *Lomariopsis* species, including African/Malagasy and Neotropical members sequenced in previous studies (Rouhan et al. 2007; Lehtonen and Cárdenas 2019), and representatives from the three remaining Lomariopsidaceae genera (Chen et al. 2017) as outgroups. Importantly, our *Lomariopsis* sampling covered almost all Asian and Oceanian species (Holttum 1932, 1939a, 1939b, 1978, Moran 2000), including four of which were phylogenetically investigated for the first time. Before this study, three species were known to be distributed in East Asia: *L.lineata* (C.Presl) Holttum (syn. *L.cochinchinensis* Fée,), *L.chinensis* Ching, and *L.boninensis*. The materials of East Asian “*L.spectabilis*” belonged to either *L.boninensis* or one of the two new species described here. Except for *L.chinensis*, all East Asian species were included in our sampling. Voucher information for all samples is provided in Appendix 1. The sequences were aligned using MUSCLE (Edgar 2004) as implemented in AliView (Larsson 2014). The alignment of every coding gene was further divided into three partitions based on the codon positions. The portions of *rps4*-*trnS* IGS (intergenic spacer), *trnL* gene, and *trnL*-*F* IGS were each treated as an independent partition as well. In the phylogenetic analyses, each partition was assigned the appropriate substitution model, which was inferred by ModelFinder (Kalyaanamoorthy et al. 2017) and using the Bayesian information criterion (BIC, Schwarz 1978).

We used IQtree 1.6.8 (Nguyen et al. 2015) to infer maximum likelihood (ML) phylogenies with 1,000 standard bootstrap replicates. The Bayesian phylogenetic analysis was performed using Mr Bayes 3.2.7 (Ronquist et al. 2012). Two simultaneous runs were carried out with four chains (5 × 10^6^ generations each). Each chain was sampled every 1,000 generations. Log likelihoods of MCMC runs were inspected in Tracer 1.6 (Rambaut and Drummond 2013) to confirm their convergence. The first 25% of the generations were conservatively discarded as burn-in.

## ﻿Results

**Figure 1.**
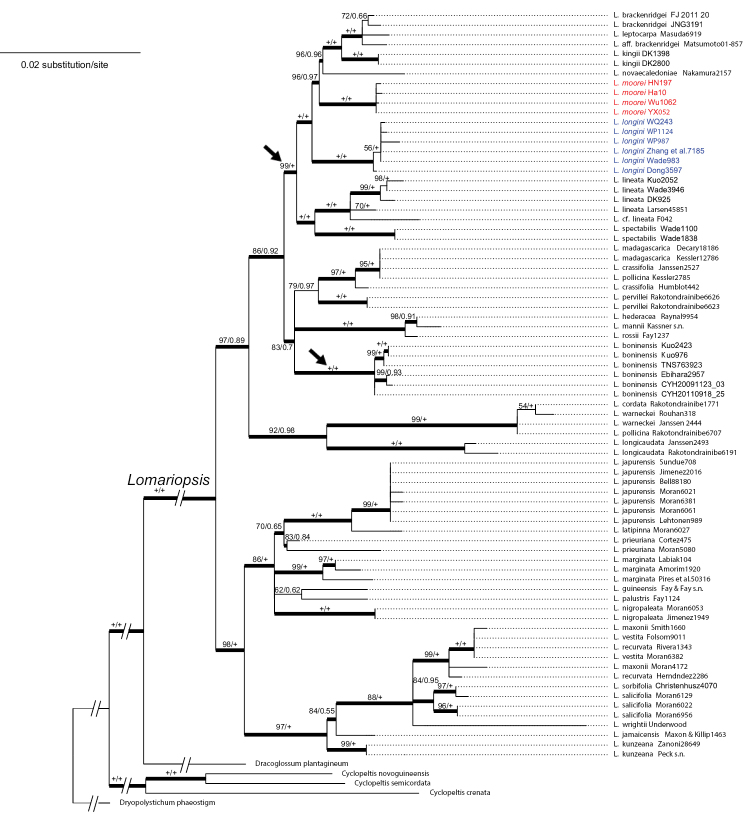
Maximum likelihood (ML) tree based on the cpDNA *rbcL* + *rps4-trnS* + *trnL-L-F* dataset. Bootstrap supports (BS) and Bayesian inference posterior probabilities (BI PP) are indicated on each branch as BS/BI PP. The arrows indicate the clades consisting of Asian and Oceanian species.

**Figure 2.**
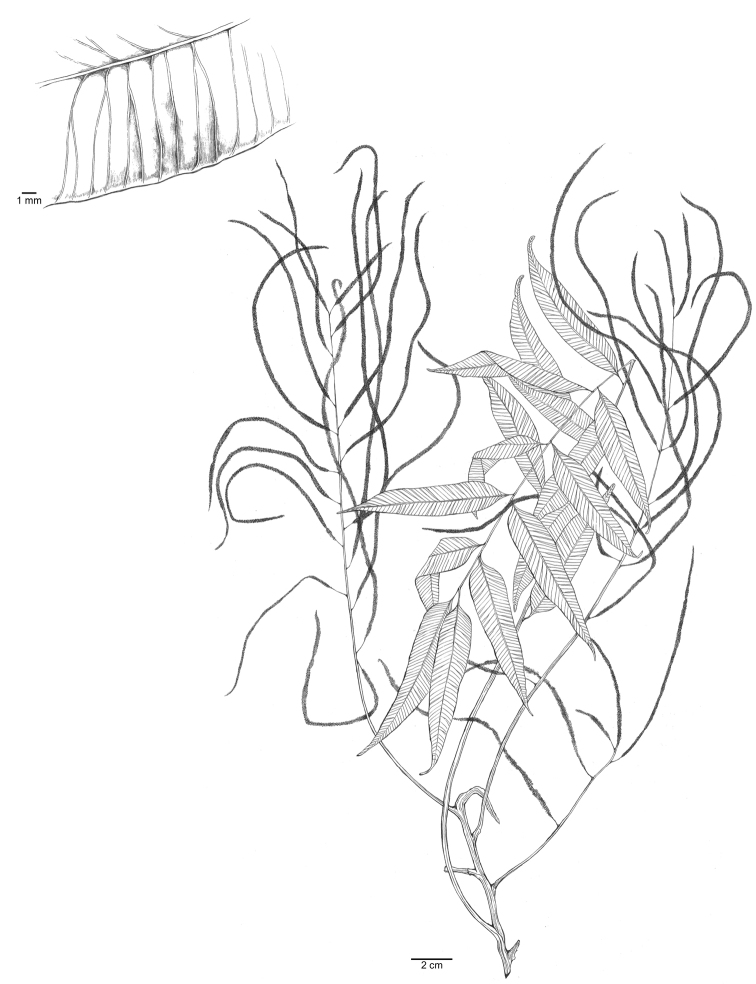
Illustration of *Lomariopsislongini* L.Y.Kuo & Y.H.Wu, sp. nov., based on the holotype *P.K. Loc* et al. 5095 (P00888363).

**Figure 3.**
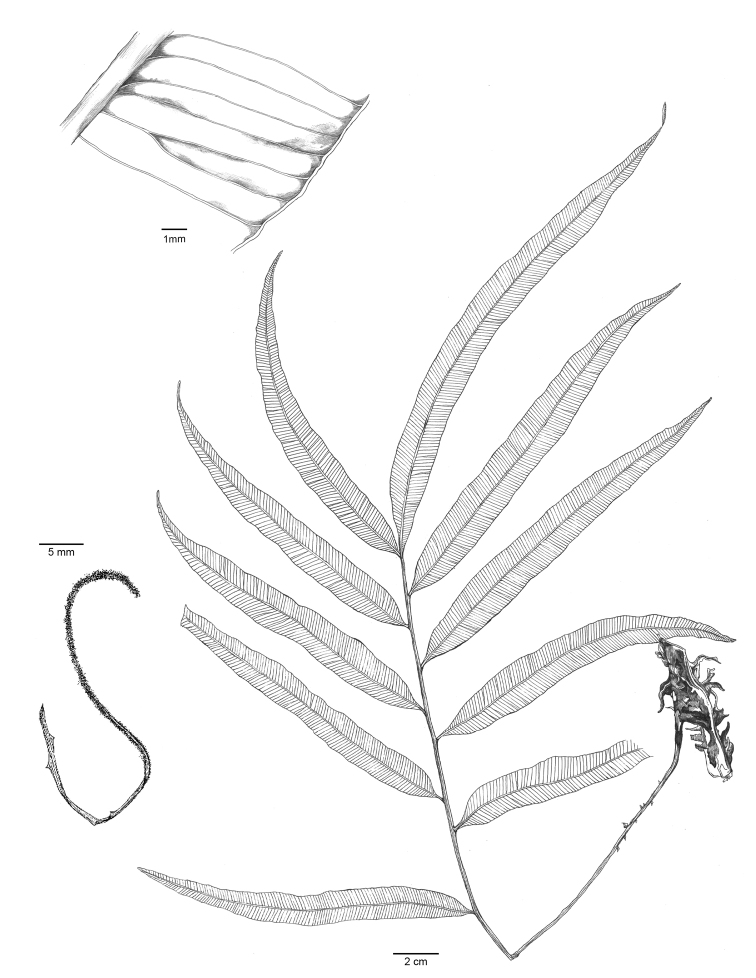
Illustration of *Lomariopsismoorei* Y.H.Wu & L.Y.Kuo, sp. nov., based on the holotype Y.H. Wu *YX052* (TAIF). A fallen fertile pinna is at the left bottom.

**Figure 4.**
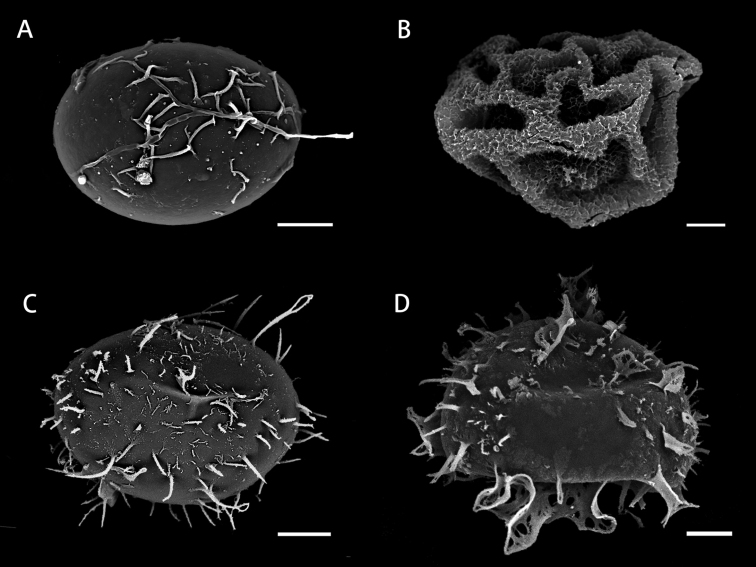
Spore perine morphology by SEM **A***Lomariopsismoorei***B***L.longini***C***L.boninensis***D***L.spectabilis*. Scale bars: 15 µm.

The combined cpDNA alignment matrix included 3,817 nucleotide sites: *rbcL* (1,431 bp), *rps4-trnS* (1,233 bp), and *trnL-L*-*F* (1,153 bp) with 27.5% of variable sites. In our phylogeny (Fig. 1), the two new species, *L.moorei* and *L.longini*, each formed a monophyletic group, and were genetically distant from *L.boninensis*, *L.spectabilis*, and other Asian and Oceanian species. The line drawings of the two new species are provided in Figs 2 and 3, and their morphological comparisons with the two Asian relatives are summarized in Table 1. Perine morphology of the four species is shown in Fig. 4.

**Table 1.** Morphological comparisons of the two new *Lomariopsis* species with their Asian morphologically similar relatives.

Characters	*L.moorei*	*L.longini*	*L.boninensis*	*L.spectabilis*
Vein apices ending at the laminar margins	yes	no	yes	yes
Widest part at upper pinna	<1/2	<1/3	<1/2	~1/2
Stipe scales	dark brown, narrowly lanceolate (usually < 2 mm wide)	dark brown, broadly lanceolate (usually >3 mm wide)	dark brown, broadly lanceolate (usually >2 mm wide)	light brown, narrowly lanceolate (usually <2 mm wide)
Fertile pinna pairs	10–14	3–9	4–16	6–14
Swollen ring on articulate at abaxial side, especially upper pinnae (Fig. 6)	obvious	obvious	inconspicuous	inconspicuous
Spore perine (Fig. 4)	spiny	folded	spiny	cristate
Spore number per sporangium	64	32	64	16 or 32

### ﻿Taxonomic treatment

#### ﻿ Lomariopsis longini

Taxon classificationPlantaePolypodialesLomariopsidaceae

L.Y.Kuo & Y.H.Wu sp. nov.

58E92A43-DA01-510D-8552-191F9C25F153

urn:lsid:ipni.org:names:77234526-1

Figs 2 , 5A

##### Diagnosis.

*Lomariopsislongini* differs from the other similar species, *L.spectabilis*, *L.boninensis*, and *L.moorei*, by its lanceolate upper sterile pinna with the widest portion occurring below the middle of the pinna, and the veins end ca. 0.5 mm before the margins.

##### Type.

Vietnam. Ha Tinh. Huong Son District: Son Kim Municipality, Rao Bun stream, 4 May 2004, *P.K. Loc 5095* (holotype: P [P00888363]! isotype: MO!).

##### Description.

Rhizomes stramineous, 0.7–1.2 cm in diam., densely scaly; rhizome scales brown (but blackened at point of attachment), lanceolate, ca. 4–9 × 1.5–3.7 mm. Fronds 1-pinnate, leathery, mature laminae pinnate, dimorphic. Sterile fronds 30–60 cm long, stipes stramineous, 10–20 cm, grooved adaxially, base with scattered scales, lateral pinnae 3–9 pairs, lanceolate, widest in the lower third, 7–16 × 1.5–1.7 cm, apex acuminate; pinna bases cuneate and decurrent, margins entire; lateral pinnae articulate to rachis, swollen ring on abaxial articulation, terminal pinna with a similar size as lateral pinnae, not articulate; upper part of rachis narrowly winged; veins free, simple or furcate, oblique, not extended to margin. Fertile laminae similar to sterile laminae but pinnae much contracted; pinnae linear, 10–20 × ca. 0.2 cm, equilateral, stalks 0.5–1.1 cm, pinna rachis articulate. Sori acrostichoid. Spores 32 per sporangium, perine with cristae.

##### Paratypes.

Vietnam. Nghe An Province: 28 Oct 2014, *L.B. Zhang, L. Zhang & N.T. Lu 7185* (CDBI, MO, VNMN); Quang Binh Province: Bo Trach District, Phong Nha-ke Bang National Park, 7 Dec 2004, *S.K. Wu and L.K. Phan WP897* (KUN); 13 Dec 2004, *WP1124* (KUN); Quang Tri Province: Dakrong District, Trieu Nguyen Commune, 4 Nov 2009, *Y.H. Chang 20091104-005* (TAIF); 2 Nov 2009, *C.W. Chen Wade 983* (TAIF); Vinh Phuc Province: Tam Dao District, Tam Dao National Park, 14 Dec 2010, *L.Y. Kuo 1862* (TAIF, VNMN). China. Yunnan Province: 25 Aug 2014, *Q. Wei WQ243* (KUN); 2 Sep 2011, *S.Y. Dong 3597* (IBSC); 28 Mar 1987, *W.M. Ju et H.C. Yan Ju and Yan 21930* (IBK).

##### Distribution.

Northern Vietnam, west southern China (Yunnan).

##### Ecology.

In shaded places, understory of evergreen broad leaf forests, below 1,000 m in elevation.

##### Etymology.

The lanceolate shape of the terminal pinnae of sterile leaves is similar to the holy lance, which is also called Lance of Longinus.

#### ﻿ Lomariopsis moorei

Taxon classificationPlantaePolypodialesLomariopsidaceae

Y.H.Wu & L.Y.Kuo sp. nov.

C72B93F6-6AA5-5043-A3E6-2EAC88138B90

urn:lsid:ipni.org:names:77234527-1

Figs 3 , 5B , 6A

##### Diagnosis.

**Figure 5.**
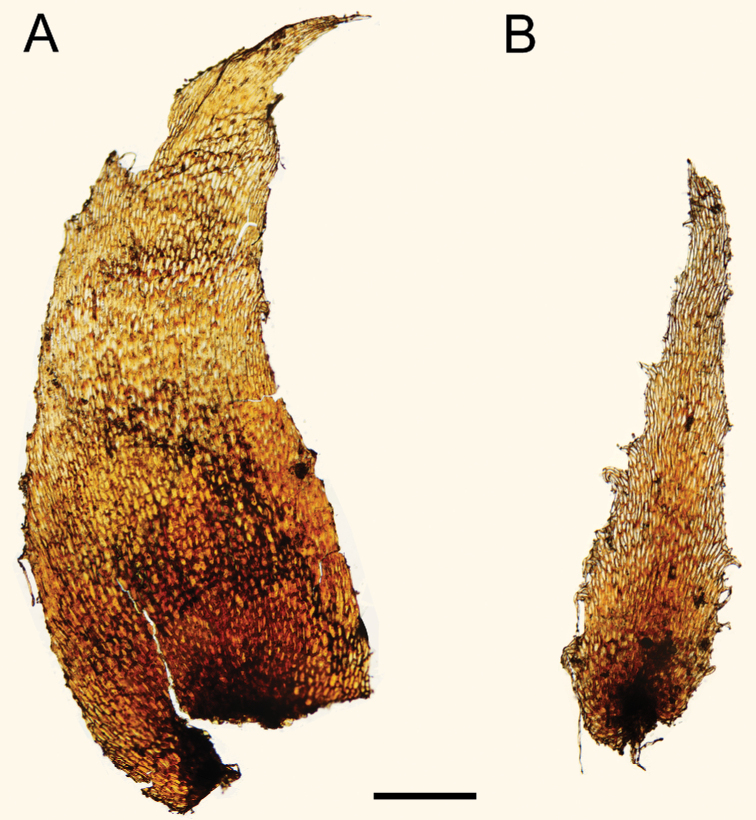
Stipe scales **A***Lomariopsislongini* (*L.Y. Kuo1862*, TAIF) **B***Lomariopsismoorei* (Y.H. Wu *YX052*, TAIF). Scale bar: 1 mm.

**Figure 6.**
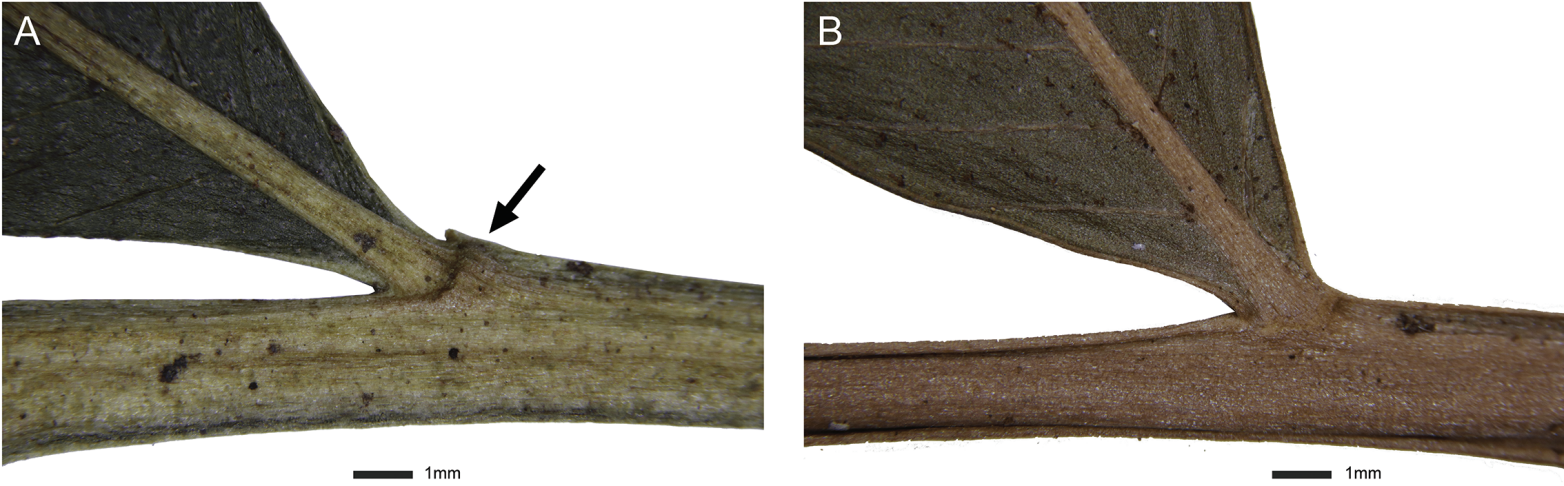
Articulation of upper pinnae (abaxial surface) to the rachis **A***Lomariopsismoorei* (Y.H. Wu YX052, TAIF) **B***Lomariopsisboninensis* (TNS790636).

*Lomariopsismoorei* is most similar to *L.boninensis*, but scales on stipes are narrower (usually < 2 mm) in *L.moorei* (Fig. 5B) and broader (usually > 2 mm) in *L.boninensis*. The swollen ring at the region of articulation on the abaxial side (especially upper pinnae) is more obvious in *L.moorei* (Table 1; Fig. 6A).

##### Type.

Taiwan. Chiayi County: Dapu Township, Zengwen Reservoir, 9 November 2020, Y.H. Wu *YX052* (holotype: TAIF! isotype: TAIF!).

##### Description.

Rhizomes rufous, 1.0–1.2 cm in diam., densely scaly; rhizome scales reddish brown (but blackened at point of attachment), narrowly lanceolate, ca. 4–6 × 0.9–2.2 mm. Fronds 1-pinnate, leathery, juvenile sterile laminae simple, shortly stalked, narrowly lanceolate, 20–25 × 1.0–1.5 cm, base narrowly cuneate, apex acute; mature lamina pinnate, dimorphic. Sterile fronds 30–50 cm long, stipes green, 10–20 cm, grooved adaxially, base with scattered scales, lateral pinnae 6–14 pairs, 1–5 cm apart, narrowly lanceolate, widest in the proximal half, 14–21 × 1.5–2.2 cm, narrowly cuneate, apex acute, base cuneate and decurrent, margin entire or slightly undulate; lateral pinnae articulate to rachis, swollen ring on abaxial articulation, terminal pinna 16–27 × 1.5–2.2 cm, not articulate; upper part of rachis narrowly winged; veins free, simple or furcate, oblique. Fertile laminae similar to sterile laminae but pinnae much contracted; pinnae linear, 10–20 × ca. 0.3 cm, equilateral, pinna rachis 0.3–0.8 cm wide, rachis articulate. Sori acrostichoid; perine consisting with glandular projections. Spores green (= chlorophyllous) and spiny, 64 per sporangium.

##### Paratypes.

Taiwan. Chiayi County: Dapu Township, Zengwen Reservoir, 9 Nov 2020, *Y.H. Wu YX053* (TAIF). China. Hainan Province: Wuzhishan City, Mt. Wuzhi National Nature Reserve, 16 July 2007, *Y.S. Chao 1211* (TAIF); Mt. Diaoluo, 27 Feb 2012, *W.H. Wu 1062* (TAIF); 21 Nov 2000, *G.M. Zhang et D. Li 117* (PE); 14 Dec 2003, *S.Y. Dong 1045* (PE).

##### Distribution.

Taiwan (Chiayi County) and China (Hainan Is.).

##### Ecology.

In shaded places, understory of evergreen broadleaf forests, below 1,000m in elevation.

##### Etymology.

The name moorei is dedicated to Dr./Mr. Shann-Jye Moore (1966–2010), an enthusiastic fern taxonomist and knowledgeable pteridologist from Taiwan. The Mr. Shann-Jye Moore Memorial Scholarship has been established by the Taiwan Society of Plant Systematics to commemorate his passions, and to support Taiwanese students studying the systematics of ferns and lycophytes.

##### Note.

We have not yet found entire sporophyll from the type locality, but fallen fertile pinnae on14 Aug 2020 (Fig. 3), which contained intact sporangia with green spores. Although mature sporophytes were found to have a restricted distribution in Taiwan, independent gametophytes of this species were found throughout Taiwan Main Is using a DNA-identification approach to survey gametophyte populations (Wu et al. in press).

## ﻿Discussion

In previous molecular phylogenies of *Lomariopsis*, none of Oceanian species were included, and *L.lineata* and *L.spectabilis* (including the misidentified *L.boninensis* and *L.longini*) were the only Asian species (Rouhan et al. 2007; Li et al. 2009; Chen et al. 2017). Here, with a comprehensive sampling in these areas, the present phylogeny (Fig. 1) provides new insights into the evolutionary relationships and systematics for *Lomariopsis* species from these areas. In the present tree, the nine Asian/Oceanian species are retrieved into two well-supported clades. The first clade consists of *L.boninensis* only, while the second clade accommodates the remaining eight species. These Asian and Oceanian clades are either nested within, or closely related to other paleotropical species (Africa and Madagascar), but their inter-clade relationships remain unclear (Fig. 1). Data from additional genetic regions will be necessary to better resolve the uncertainties of these nodes, and hence to confirm biogeographical origin(s) of Asian/Oceanian taxa. Among all six described East Asian species, *L.chinensis* is the only one missing from the current phylogeny. To the best of our knowledge, this species has only been collected once, as the type collections. Despite the lack of phylogenetic information, *L.chinensis* is morphologically unique in the genus and easily distinguished from other *Lomariopsis* species because of its reticulate leaf venation.

*Lomariopsis* species diversity in Asia and Oceania could still be underestimated, and more undocumented species could be eventually revealed by phylogenetic analyses using multiple specimens in each morphologically-defined species, similar to the case of discovering the two new species here described. Indeed, *L.moorei* and *L.longini*, together with *L.boninensis*, are genetically distant taxa in East Asia even if all three have been long misidentified and confused under a single name of the South East Asian species, *L.spectabilis*, due to their overall similar morphology (DeVol and Kuo 1975; Tsai and Shieh 1994; Iwatsuki et al. 1995; Yang and Liu 2002; Li et al. 2009; Phan 2010; Xing et al. 2013; Knapp 2014; Chen et al. 2017; Ebihara 2017; TPG 2019). However, clear molecular phylogenetic results spurred us to seek other characters supporting the distinction between these lineages, and these actual species now can be identified based on microscopic characters (Table 1). These characters include perine ornamentation, which has been revealed to have highly diversified forms in *Lomariopsis* (Rouhan et al. 2007). Additionally, we found that the spore number per sporangium varies among these species, which can also help in distinguishing species. However, unlike most cases in ferns of the Polypodiales, such a reduction in the number of spores in sporangia (e.g., 64 to 32) may not represent a reproductive switch to apomixis for Lomariopsidaceae (Chen et al. 2017). Further cytological investigations, e.g., through flow cytometry to infer both spore vs. leaf genome sizes (Kuo et al. 2017), are necessary to clarify whether changes in the two phenomena (i.e., spore number per sporangium and reproductive mode) are linked in these *Lomariopsis* species.

### ﻿Key to *Lomariopsislongini*, *L.moorei*, *L.spectabilis*, and other morphologically close species in East Asia

**Table:** 

1	Sterile lateral pinnae with lateral veins spreading (borne at nearly right angles to the pinna rachis), free, occasionally anastomosing	***L.chinensis***
–	Sterile lateral pinnae with veins oblique, free	**2**
2	Sterile lateral pinnae, abruptly narrowed to a caudate apex (2–3 cm long)	***L.lineata***
–	Sterile lateral pinnae with acuminate apex	**3**
3	Sterile lateral pinnae lanceolate, widest in the lower third	***L.longini***
–	Sterile lateral pinnae narrowly lanceolate, widest in the middle	**4**
4	Sterile lateral pinnae with pinna stalks (0.3–0.7 cm long), base equilateral	***L.spectabilis***
–	Sterile lateral pinnae with, pinna subsessile, base cuneate and decurrent	**5**
5	Swollen ring inconspicuous on abaxial articulation (especially upper pinnae), scales on the stipes broadly lanceolate (> 2 mm wide)	***L.boninensis***
–	Swollen ring obvious on abaxial articulation side (especially upper pinnae), scales on the stipes narrowly lanceolate (< 2 mm wide)	***L.moorei***

## Supplementary Material

XML Treatment for Lomariopsis longini

XML Treatment for Lomariopsis moorei

## ﻿Appendix 1

**Table:** Voucher information and GenBank accession numbers. –, sequences not available. *sequences obtained in this study.

Species name	Locality	Voucher (Herbarium)	GenBank accession number		
			*rbcL*	*rps4-trnS*	*trnL-L-F*
*Cyclopeltiscrenata*	China, Hainan	Wu935 (TAIF)	OL420736*	OL473642*	OL473665*
*Cyclopeltisnovoguineensis*	Solomon Islands	SITW068 (TAIF)	KY397974	KY397978	KY397970
*Cyclopeltissemicordata*	Costa Rica	CJR et al. 08-195 (DUKE)	EF463234	KY397977	KY397969
*Dracoglossumplantagineum*	Guadeloupe	Christenhusz 4065 (TUR)	KY397975	KC914565	KY397971
*Dryopolystichumphaeostigma*	Solomon Islands	SITW10443 (BSIP, TAIF, TNM)	KY397972	KY397976	KY397968
Lomariopsisaff.brackenridgei	Vanuatu, Espiritu Santo Is.	Matsumoto 01-857 (TNS)	-	-	OL473666*
*Lomariopsisboninensis*	Japan, Bonin Is.	TNS 763923 (TNS)	AB575226	OL473643*	OL473667*
*Lomariopsisboninensis*	Japan, Ishigaki Is.	Ebihara 2957 (TNS)	OL420737*	OL473644*	OL473668*
*Lomariopsisboninensis*	Taiwan, Orchid Is.	CYH20091123_03 (TAIF)	OL420738*	OL473645*	OL473669*
*Lomariopsisboninensis*	Taiwan, Orchid Is.	CYH20110918_25 (TAIF)	-	OL473646*	OL473670*
*Lomariopsisboninensis*	Taiwan	Kuo 2423 (TAIF)	OL420739*	OL473647*	OL473671*
*Lomariopsisboninensis*	Taiwan	Kuo 976 (TAIF)	OL420740*	OL473648*	OL473672*
*Lomariopsisbrackenridgei*	Fiji	FJ_2011_207 (WELT)	OL420741*	OL473649*	OL473673*
*Lomariopsisbrackenridgei*	French Polynesia, Moorea	JNG3191 (UC)	-	-	OL473674*
Lomariopsiscf.lineata	Unknown Origin	F042 (SING)	OL420742*	AM947063	AM946393
*Lomariopsisjapurensis*	Unknown Origin	Lehtonen 989 (TUR)	MK705752	MK705752	MK705752
*Lomariopsiskingii*	Indonesia, Bacan	DK1398 (UC)	OL420743*	OL473650*	OL473675*
*Lomariopsiskingii*	New Guinea	DK2800 (UC)	OL420744*	OL473651*	OL473676*
*Lomariopsisleptocarpa*	Micronesia	Masuda 6919 (TNS)	OL420745*	OL473652*	OL473677*
*Lomariopsislineata*	Indonesia, Sulawesi	DK925 (UC)	OL420746*	OL473653*	OL473678*
*Lomariopsislineata*	Philippines	Kuo 2052 (TAIF)	OL420747*	OL473654*	OL473679*
*Lomariopsislineata*	Philippines	Wade 3946 (TAIF)	-	-	OL473680*
*Lomariopsisnovaecaledoniae*	New Caledonia	Nakamura 2157 (TNS)	-	OL473655*	OL473681*
*Lomariopsissorbifolia*	Guadeloupe	Christenhusz 4070 (TUR)	EF463236	OL473656*	OL473682*
*Lomariopsislongini*	China, Yunnan	Dong 3597 (IBSC)	OL420748*	OL473657*	OL473683*
*Lomariopsislongini*	China, Yunnan	WQ243 (KUN)	OL420749*	OL473658*	OL473684*
*Lomariopsislongini*	Vietnam	Wade983 (TAIF)	OL420750*	OL473659*	OL473685*
*Lomariopsislongini*	Vietnam	Zhang et al.7185	KU605187	KU605086	KU605107
*Lomariopsislongini*	Vietnam	WP897 (KUN)	-	-	OL473686*
*Lomariopsislongini*	Vietnam	WP1124 (KUN)	-	-	OL473687*
*Lomariopsismoorei*	Taiwan	YX052 (TAIF)	OL420751*	OL473660*	OL473688*
*Lomariopsismoorei*	China, Hainan	HN197 (PE)	OL420752*	OL473661*	OL473689*
*Lomariopsismoorei*	Taiwan	Ha 10**	-	-	OL473690*
*Lomariopsismoorei*	China, Hainan	Wu 1062 (TAIF)	OL420753*	OL473662*	OL473691*
*Lomariopsisspectabilis*	Indonesia, Java	Wade 1100 (TAIF)	OL420754*	OL473663*	OL473692*
*Lomariopsisspectabilis*	Indonesia, Java	Wade 1838 (TAIF)	OL420755*	OL473664*	OL473693*
*Lomariopsiscordata*	Madagascar	Rakotondrainibe 1771 (P)	-	-	DQ396558
*Lomariopsiscrassifolia*	Madagascar	Janssen et al. 2527 (P)	-	-	DQ396602
*Lomariopsiscrassifolia*	Madagascar	Humblot 442 (P)	-	-	DQ396559
*Lomariopsisguineensis*	Sierra Leone	Fay & Fay s.n., in 1985 (NY)	-	-	DQ396560
*Lomariopsishederacea*	Cameroon	Raynal 9954 (P)	-	-	DQ396561
*Lomariopsisjamaicensis*	Jamaica	Maxon & Killip 1463 (NY)	-	-	DQ396562
*Lomariopsisjapurensis*	Ecuador	Moran 6021 (NY, QCA, QCNE)	-	-	DQ396566
*Lomariopsisjapurensis*	Ecuador	Moran 6061 (NY, QCA, QCNE)	-	-	DQ396565
*Lomariopsisjapurensis*	Costa Rica	Moran 6381(CR, INB, NY)	-	-	DQ396567
*Lomariopsisjapurensis*	Bolivia	Sundue 708 (LPB, NY, USZ)	-	-	DQ396563
*Lomariopsisjapurensis*	Peru	Bell 88180 (NY)	-	-	DQ396564
*Lomariopsisjapurensis*	Bolivia	Jimenez 2016 (LPB, NY)	-	-	DQ396568
*Lomariopsiskunzeana*	Haiti	Zanoni 28649 (EHH, NY)	-	-	DQ396570
*Lomariopsiskunzeana*	United States	Peck s.n. (NY)	-	-	DQ396569
*Lomariopsislatipinna*	Ecuador	Moran 6027 (NY, QCA, QCNE, TUR)	-	-	DQ396571
*Lomariopsislineata*	Thailand	Larsen 45851 (AAU, NY)	-	-	DQ396572
*Lomariopsislongicaudata*	Madagascar	Janssen 2493 (P)	-	-	DQ396574
*Lomariopsislongicaudata*	Madagascar	Rakotondrainibe 6191 (P)	-	-	DQ396573
*Lomariopsismadagascarica*	Madagascar	Kessler 12786 (NY)	-	-	DQ396575
*Lomariopsismadagascarica*	Madagascar	Decary 18186 (P)	-	-	DQ396576
*Lomariopsismannii*	Democratic Republic Of Congo	Kassner s.n., in 1914 (P)	-	-	DQ396577
*Lomariopsismarginata*	Brazil	Pires et al. 50316 (NY)	-	-	DQ396579
*Lomariopsismarginata*	Brazil	Amorim 1920 (CEPEC, NY)	-	-	AY540045
*Lomariopsismarginata*	Brazil	Labiak 104 (NY, UPCB)	-	-	DQ396578
*Lomariopsismaxonii*	Costa Rica	Moran 4172 (CR, NY, UC)	-	-	DQ396580
*Lomariopsismaxonii*	Costa Rica	Smith 1660 (CR, NY, UC)	-	-	DQ396581
*Lomariopsisnigropaleata*	Ecuador	Moran 6053 (NY)	-	-	DQ396584
*Lomariopsisnigropaleata*	Bolivia	Jimenez 1949 (LPB, NY)	-	-	DQ396583
*Lomariopsispalustris*	Sierra Leone	Fay 1124 (NY)	-	-	DQ396585
*Lomariopsispervillei*	Madagascar	Rakotondrainibe et al. 6626 (P)	-	-	DQ396586
*Lomariopsispervillei*	Madagascar	Rakotondrainibe et al. 6623 (P)	-	-	DQ396587
*Lomariopsiswarneckei*	Comoros	Rakotondrainibe et al. 6707 (P)	-	-	DQ396588
*Lomariopsispollicina*	Madagascar	Kessler 12785 (GOET, NY)	-	-	DQ396589
*Lomariopsisprieuriana*	Venezuela	Cortez 475 (NY, VEN)	-	-	DQ396591
*Lomariopsisprieuriana*	Panama	Moran 5080 (MO, NY, PMA)	-	-	DQ396590
*Lomariopsisrecurvata*	Mexico	Rivera 1343 (NY)	-	-	DQ396592
*Lomariopsisrecurvata*	Mexico	Hernandez 2286 (NY)	-	-	DQ396593
*Lomariopsisrossii*	Liberia	Fay 1237 (NY)	-	-	DQ396594
*Lomariopsissalicifolia*	Ecuador	Moran 6022 (AAU, NY, QCA, QCNE, TUR)	-	-	DQ396597
*Lomariopsissalicifolia*	Ecuador	Moran 6129 (NY, QCA, QCNE)	-	-	DQ396595
*Lomariopsissalicifolia*	Ecuador	Moran 6956 (NY, QCA, QCNE)	-	-	DQ396596
*Lomariopsiswarneckei*	Madagascar	Janssen et al. 2444 (P)	-	-	DQ396601
*Lomariopsiswarneckei*	Madagascar	Rouhan et al. 318 (P)	-	-	DQ396603
*Lomariopsisvestita*	Costa Rica	Moran 6382 (CR, INB, NY, USJ)	-	-	DQ396598
*Lomariopsisvestita*	Costa Rica	Folsom 9011 (NY)	-	-	DQ396599
*Lomariopsiswrightii*	Cuba	Underwood 948 (NY)	-	-	DQ396600
